# Biomechanics in DALK: Big bubble vs Manual lamellar dissection

**DOI:** 10.5935/0004-2749.20200076

**Published:** 2020

**Authors:** Akilesh Gokul, Lize Angelo, Hans Vellara, Mohammed Ziaei

**Affiliations:** 1 Department of Ophthalmology, New Zealand National Eye Centre, Faculty of Medical and Health Sciences, University of Auckland, Auckland, New Zealand

Dear Editor:

We read with interest the results of the study by Akdemir et al, which compared the biomechanical properties of eyes undergoing big bubble deep anterior lamellar keratoplasty (DALK) with those undergoing predescematic or manual DALK^(1)^. Although the results are of interest, several issues require clarification.

In predescematic DALK, a residual layer of host posterior stroma is left intact, which allows for wound healing to occur at the deep lamellar interface as well as at the peripheral wound edge^(2)^. Thus, it is conceivable that a very thin residual stromal bed thickness after DALK could allow for biomechanical properties similar to that of a BB DALK and that a thicker residual stromal bed could provide additional biomechanical support. However, the authors do not look at this variable in their study when comparing the two groups but rather report on postoperative corneal thickness. In our recent study investigating the biomechanical properties of predescematic DALK, we demonstrated a correlation between residual central host thickness and biomechanical properties of the cornea^(3)^.

In addition, the authors fail to report their postoperative steroid regimen and indicate whether there was any disparity in steroid use between the two groups. This is important because steroid use has previously been reported to prolong the instability induced by corneal incisions^(4)^.

Finally, although the authors report the sutures were removed in all patients at least 3 months before their biomechanical evaluation, they do not present the mean time from suture removal, and it is possible that a disparity between the lengths of time that the sutures remained *in situ* could have altered the wound healing response of the graft-host junction.

## References

[1] Akdemir MO, Acar BT, Acar S. (2020). Biomechanics in DALK: Big bubble vs manual lamellar
dissection. Arq Bras Oftalmol.

[2] Keane M, Coster D, Ziaei M (2014). Deep anterior lamellar keratoplasty versus penetrating
keratoplasty for treating keratoconus. Cochrane Database Syst Rev.

[3] Ziaei M, Vellara HR, Gokul A (2019). Comparison of corneal biomechanical properties following
penetrating keratoplasty and deep anterior lamellar keratoplasty for
keratoconus. Clin Exp Ophthalmol.

[4] McCarey BE, Napalkov JA, Pippen PA (1995). Corneal wound healing strength with topical antiinflammatory
drugs. Cornea.

