# Supplementary material for: Biomechanics in DALK: Big bubble vs Manual lamellar dissection
Source: Arq Bras Oftalmol. 2020 Jul-Aug;83(4):354–5. doi: 10.5935/0004-2749.20200076 (PMC11826587; doi:10.5935/0004-2749.20200076)
Supplement: Related texts

# Reply: Biomechanics in DALK: Big bubble vs Manual lamellar dissection

**Authors:** Mehmet Orcun Akdemir, Banu Torun Acar, Suphi Acar

**Affiliations:** 1 Department of Ophthalmology, School of Medicine, Bulent Ecevit University, Zonguldak, Turkey; 2 Haydarpasa Numune Research and Education Hospital, Istanbul, Turkey

The questions and comments from Ziaei et al. provide us an opportunity to discuss the unclear points of our manuscript regarding the comparison of biomechanics in deep anterior lamellar keratoplasty (DALK). As they pointed out in their recent study investigating the biomechanical properties of predescematic DALK, they concluded that neither penetrating keratoplasty (PKP) nor the DALK technique used in keratoconus completely restored the biomechanical properties of the cornea to the level of healthy corneas. However, as compared with DALK, PKP resulted in a greater number of parameters that were significantly different from healthy corneas^(1)^. We also compared the two techniques in patients with keratoconus and found results similar to those of Ziaei et al., in that the PKP technique resulted in weaker corneal biomechanical properties. However, we found different results between corneas undergoing DALK surgery and normal corneas. In our study, DALK surgery resulted in similar corneal hysteresis results as those of healthy corneas^(2)^. These results are not consistent with the study by Ziaei et al. In light of our results, we did not include the residual thickness of the stroma when comparing the DALK techniques. Moreover, our aim was primarily to evaluate the results of DALK surgery in cases of clear Descemet’s membrane and with a residual stromal bed.

Zeiei et al. also focused their attention on reporting the postoperative steroid regimen and whether any disparity existed between the two groups in steroid use. It is clear that the postoperative steroid regimen was different between the PKP and DALK surgeries. This is attributed to the difference in corneal wound healing, endothelial rejection rates, and so forth^(3)^. We did not use different steroid treatment protocols between patients with clear Descemet’s membrane and residual stromal bed in DALK surgery. In addition, we believe that residual stroma had no effect on steroid use in cases in which a big bubble cannot be created, because adequate steroid treatment was administered in both groups. We have applied the same postoperative steroid regimen since 2008, and we have not seen the need to change this protocol in patients with DALK since then.

The advantages of DALK surgery over PKP are obvious and include the fact that topical corticosteroids can usually be discontinued earlier after DALK, DALK may have superior resistance to rupture of the globe after blunt trauma, and sutures can be removed earlier with DALK^(3)^. In their clinical and confocal study, Abdelkader and Kaufman found that progressive reduction of keratocyte brightness and reflectivity occurred in patients undergoing DALK surgery. In the predescemetic group, keratocyte morphology and reflectivity returned to normal by 10 to 12 weeks, whereas this normalization process took 4 to 6 weeks in the descemetic group^(4)^. In light of this study, the initial healing of the eye should have been completed by 3 months postgraft. Therefore, we prefer to set the minimum suture removal time as 3 months.

## References

[1] Ziaei M, Vellara HR, Gokul A (2019). Comparison of corneal biomechanical properties following
penetrating keratoplasty and deep anterior lamellar keratoplasty for
keratoconus. Clin Exp Ophthalmol.

[2] Torun Acar Banu, Orcun Akdemir Mehmet, Suphi Acar (2013). Corneal biomechanical properties in eyes with no previous
surgery, with previous penetrating keratoplasty and with deep anterior
lamellar keratoplasty. Japan J Ophthalmol.

[3] Reinhart William J, Musch DC, Jacops DC (2011). Deep anterior lamellar keratoplasty as an alternative to
penetrating keratoplasty: a report by the American Academy of
Ophthalmology. Ophthalmology.

[4] Abdelkader A, Kaufman HE. (2011). Descemetic versus pre-descemetic lamellar keratoplasty:
clinical and confocal study. Cornea.
